# 8-Phenyl-10-oxa-8-aza­tricyclo­[4.3.0.1^2,5^]decane-7,9-dione

**DOI:** 10.1107/S1600536809000282

**Published:** 2009-01-10

**Authors:** Wen-Zhong Zhu, Qiu-Yue Lin

**Affiliations:** a Zhejiang Key Laboratory for Reactive Chemistry on Solid Surfaces, Institute of Physical Chemistry, Zhejiang Normal University, Jinhua, Zhejiang 321004, People’s Republic of China, and, College of Chemistry and Life Science, Zhejiang Normal University, Jinhua 321004, Zhejiang, People’s Republic of China

## Abstract

The reaction of aniline with norcantharidin produced the imide title compound, C_14_H_13_NO_3_, which shows no significant hydrogen bonds in the crystal structure. The dihedral angle between the phenyl and pyrrolidine rings is 48.48 (6)°.

## Related literature

For the use of norcantharidin in synthesis see: Hill *et al.* (2007 ▶). For background, see: Wang (1989 ▶).
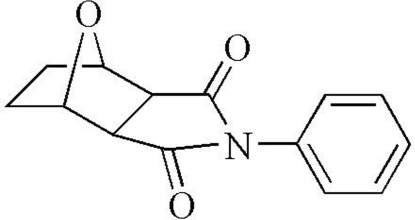


## Experimental

### 

#### Crystal data

C_14_H_13_NO_3_
*M* *_r_* = 243.25
Monoclinic, 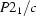

*a* = 9.5914 (4) Å
*b* = 8.4345 (3) Å
*c* = 14.4101 (6) Å
β = 93.468 (3)°
*V* = 1163.62 (8) Å^3^
*Z* = 4
Mo *K*α radiation
μ = 0.10 mm^−1^
*T* = 296 (2) K
0.32 × 0.25 × 0.04 mm

#### Data collection

Bruker APEXII CCD area-detector diffractometer
Absorption correction: multi-scan (*SADABS*; Sheldrick, 1996 ▶) *T*_min_ = 0.971, *T*_max_ = 0.996
18085 measured reflections
2699 independent reflections
1898 reflections with *I* > 2σ(*I*)
*R*_int_ = 0.041

#### Refinement

*R*[*F* ^2^ > 2σ(*F* ^2^)] = 0.041
*wR*(*F* ^2^) = 0.177
*S* = 0.61
2699 reflections
163 parameters
H-atom parameters constrained
Δρ_max_ = 0.16 e Å^−3^
Δρ_min_ = −0.17 e Å^−3^

### 

Data collection: *APEX2* (Bruker, 2004 ▶); cell refinement: *SAINT* (Bruker, 2004 ▶); data reduction: *SAINT*; program(s) used to solve structure: *SHELXS97* (Sheldrick, 2008 ▶); program(s) used to refine structure: *SHELXL97* (Sheldrick, 2008 ▶); molecular graphics: *SHELXTL* (Sheldrick, 2008 ▶); software used to prepare material for publication: *SHELXL97*.

## Supplementary Material

Crystal structure: contains datablocks I, global. DOI: 10.1107/S1600536809000282/at2701sup1.cif

Structure factors: contains datablocks I. DOI: 10.1107/S1600536809000282/at2701Isup2.hkl

Additional supplementary materials:  crystallographic information; 3D view; checkCIF report

## Figures and Tables

**Table 1:** Hydrogen-bond geometry (Å, °)

*D*—H⋯*A*	*D*—H	H⋯*A*	*D*⋯*A*	*D*—H⋯*A*
C10—H10*B*⋯O2^i^	0.97	2.59	3.502 (3)	156

Symmetry code: (i) 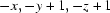.

**Fig. 1.**
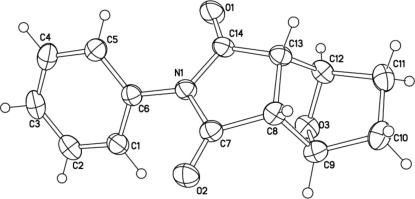
A view of the molecule of (I) showing the atom-labelling scheme with displacement ellipsoids drawn at the 30% probability.

## supplementary crystallographic information

### Comment

Norcantharidin are a variety of pharmacologically important compounds such as protein kinase inhibitors and antitumor properties (Wang, 1989). We have designed, synthesized and crystallized several norcantharidin derivatives to study their anticancer properties. In order to study on the relationship between the activity of norcantharidin and the importance of aromatic ring linked to the carboxyl, the norcantharidin derivative was synthesized and its crystal structure is reported here.

X-ray crystallography confirmed the molecular structure and the atom connectivity for the title compound, as illustrated in Fig. 1. In the compound, the dihedral angle between the mean planes of pyrrolidine (C7/C8/C13/C14/N1) rings and the phenyl (C1—C6) is 48.48 (6)°. It exhibits no unusual crystal packing features, and each molecule acts as a donor and acceptor for one C10—H10B···O2 weak intermolecular hydrogen bonds.

### Experimental

The title compound was synthesized by the condensation of norcantharidin (1 mmol) with aniline (1 mmol) in DMF (10 mL). After refluxing for 3 h, the reaction mixture was left to stand for two weeks, colourless crystals were isolated.

### Refinement

The H atoms bonded to C atoms were positioned geometrically and refined using a riding model [C—H = 0.93 - 0.98 Å, *U*_iso_(H) = 1.2*U*_eq_(C)].

### Crystal data

**Table:**

C_14_H_13_NO_3_	*F*(000) = 512
*M**_r_* = 243.25	*D*_x_ = 1.389 Mg m^−^^3^*D*_m_ = 1.389 Mg m^−^^3^*D*_m_ measured by not measured
Monoclinic, *P*2_1_/*c*	Mo *K*α radiation, λ = 0.71073 Å
Hall symbol: -P 2ybc	Cell parameters from 3324 reflections
*a* = 9.5914 (4) Å	θ = 2.1–27.7°
*b* = 8.4345 (3) Å	µ = 0.10 mm^−^^1^
*c* = 14.4101 (6) Å	*T* = 296 K
β = 93.468 (3)°	Sheet, colourless
*V* = 1163.62 (8) Å^3^	0.32 × 0.25 × 0.04 mm
*Z* = 4	

### Data collection

**Table:**

Bruker SMART CCD area-detector diffractometer	2699 independent reflections
Radiation source: fine-focus sealed tube	1898 reflections with *I* > 2σ(*I*)
graphite	*R*_int_ = 0.041
φ and ω scans	θ_max_ = 27.7°, θ_min_ = 2.1°
Absorption correction: multi-scan (*SADABS*; Sheldrick, 1996)	*h* = −12→12
*T*_min_ = 0.971, *T*_max_ = 0.996	*k* = −11→10
18085 measured reflections	*l* = −18→18

### Refinement

**Table:**

Refinement on *F*^2^	Primary atom site location: structure-invariant direct methods
Least-squares matrix: full	Secondary atom site location: difference Fourier map
*R*[*F*^2^ > 2σ(*F*^2^)] = 0.041	Hydrogen site location: inferred from neighbouring sites
*wR*(*F*^2^) = 0.177	H-atom parameters constrained
*S* = 0.61	*w* = 1/[σ^2^(*F*_o_^2^) + (0.1908*P*)^2^ + 0.6671*P*] where *P* = (*F*_o_^2^ + 2*F*_c_^2^)/3
2699 reflections	(Δ/σ)_max_ < 0.001
163 parameters	Δρ_max_ = 0.16 e Å^−^^3^
0 restraints	Δρ_min_ = −0.17 e Å^−^^3^

### Special details

**Table:**

Geometry. All e.s.d.'s (except the e.s.d. in the dihedral angle between two l.s. planes) are estimated using the full covariance matrix. The cell e.s.d.'s are taken into account individually in the estimation of e.s.d.'s in distances, angles and torsion angles; correlations between e.s.d.'s in cell parameters are only used when they are defined by crystal symmetry. An approximate (isotropic) treatment of cell e.s.d.'s is used for estimating e.s.d.'s involving l.s. planes.
Refinement. Refinement of *F*^2^ against ALL reflections. The weighted *R*-factor *wR* and goodness of fit *S* are based on *F*^2^, conventional *R*-factors *R* are based on *F*, with *F* set to zero for negative *F*^2^. The threshold expression of *F*^2^ > σ(*F*^2^) is used only for calculating *R*-factors(gt) *etc*. and is not relevant to the choice of reflections for refinement. *R*-factors based on *F*^2^ are statistically about twice as large as those based on *F*, and *R*- factors based on ALL data will be even larger.

### Fractional atomic coordinates and isotropic or equivalent isotropic displacement parameters (Å^2^)

**Table:**

	*x*	*y*	*z*	*U*_iso_*/*U*_eq_	
N1	0.10268 (15)	0.22038 (16)	0.69370 (9)	0.0418 (4)	
O1	−0.04620 (15)	0.1156 (2)	0.79702 (10)	0.0655 (4)	
O2	0.19084 (14)	0.33610 (17)	0.56481 (9)	0.0579 (4)	
O3	−0.13027 (14)	0.06451 (14)	0.54810 (10)	0.0526 (4)	
C1	0.32246 (19)	0.0814 (2)	0.68510 (13)	0.0513 (4)	
H1A	0.3026	0.0665	0.6217	0.062*	
C2	0.4439 (2)	0.0209 (3)	0.72752 (17)	0.0620 (5)	
H2A	0.5055	−0.0358	0.6927	0.074*	
C3	0.4743 (2)	0.0442 (3)	0.82150 (17)	0.0646 (6)	
H3A	0.5562	0.0034	0.8499	0.078*	
C4	0.3832 (2)	0.1278 (2)	0.87295 (15)	0.0599 (5)	
H4A	0.4042	0.1435	0.9361	0.072*	
C5	0.2603 (2)	0.1891 (2)	0.83178 (13)	0.0493 (4)	
H5A	0.1989	0.2457	0.8668	0.059*	
C6	0.23040 (18)	0.16445 (19)	0.73715 (12)	0.0421 (4)	
C7	0.09220 (19)	0.29901 (19)	0.60787 (11)	0.0432 (4)	
C8	−0.05956 (18)	0.32348 (19)	0.57997 (11)	0.0432 (4)	
H8A	−0.0820	0.4349	0.5668	0.052*	
C9	−0.1114 (2)	0.2122 (2)	0.50018 (13)	0.0502 (4)	
H9A	−0.0485	0.2054	0.4492	0.060*	
C10	−0.2605 (2)	0.2614 (3)	0.46982 (14)	0.0592 (5)	
H10A	−0.2906	0.2148	0.4104	0.071*	
H10B	−0.2696	0.3758	0.4659	0.071*	
C11	−0.3429 (2)	0.1933 (2)	0.54908 (16)	0.0585 (5)	
H11A	−0.3931	0.2756	0.5802	0.070*	
H11B	−0.4082	0.1120	0.5267	0.070*	
C12	−0.22552 (19)	0.1238 (2)	0.61262 (14)	0.0488 (4)	
H12A	−0.2570	0.0432	0.6556	0.059*	
C13	−0.13944 (18)	0.25713 (19)	0.66061 (11)	0.0430 (4)	
H13A	−0.1973	0.3369	0.6894	0.052*	
C14	−0.02839 (18)	0.1892 (2)	0.72702 (12)	0.0449 (4)	

### Atomic displacement parameters (Å^2^)

**Table:**

	*U*^11^	*U*^22^	*U*^33^	*U*^12^	*U*^13^	*U*^23^
N1	0.0422 (8)	0.0437 (7)	0.0400 (7)	0.0010 (6)	0.0060 (6)	0.0082 (6)
O1	0.0549 (8)	0.0882 (11)	0.0546 (8)	0.0025 (7)	0.0131 (6)	0.0281 (7)
O2	0.0542 (8)	0.0651 (8)	0.0555 (8)	−0.0097 (6)	0.0127 (6)	0.0158 (6)
O3	0.0538 (7)	0.0369 (6)	0.0678 (8)	0.0025 (5)	0.0099 (6)	−0.0059 (5)
C1	0.0465 (10)	0.0543 (10)	0.0534 (10)	−0.0008 (8)	0.0072 (8)	−0.0011 (8)
C2	0.0440 (10)	0.0628 (12)	0.0799 (14)	0.0028 (9)	0.0097 (9)	0.0026 (10)
C3	0.0417 (10)	0.0654 (13)	0.0851 (15)	−0.0065 (9)	−0.0098 (10)	0.0115 (11)
C4	0.0588 (12)	0.0611 (12)	0.0580 (11)	−0.0136 (9)	−0.0121 (9)	0.0061 (9)
C5	0.0533 (10)	0.0465 (9)	0.0481 (9)	−0.0053 (8)	0.0025 (8)	0.0012 (7)
C6	0.0414 (8)	0.0397 (8)	0.0456 (9)	−0.0038 (6)	0.0042 (7)	0.0050 (7)
C7	0.0486 (9)	0.0398 (8)	0.0415 (8)	−0.0053 (7)	0.0059 (7)	0.0044 (6)
C8	0.0490 (9)	0.0359 (8)	0.0444 (9)	−0.0014 (7)	0.0015 (7)	0.0053 (6)
C9	0.0568 (11)	0.0490 (9)	0.0450 (9)	−0.0032 (8)	0.0053 (8)	−0.0019 (7)
C10	0.0640 (13)	0.0534 (10)	0.0579 (11)	−0.0032 (9)	−0.0139 (9)	−0.0036 (9)
C11	0.0462 (10)	0.0540 (11)	0.0741 (13)	0.0006 (8)	−0.0060 (9)	−0.0081 (9)
C12	0.0434 (9)	0.0398 (8)	0.0639 (11)	−0.0005 (7)	0.0090 (8)	0.0043 (8)
C13	0.0441 (9)	0.0396 (8)	0.0459 (9)	0.0050 (7)	0.0082 (7)	0.0018 (7)
C14	0.0434 (9)	0.0472 (9)	0.0450 (9)	0.0024 (7)	0.0100 (7)	0.0044 (7)

### Geometric parameters (Å, °)

**Table:**

N1—C14	1.398 (2)	C5—H5A	0.9300
N1—C7	1.402 (2)	C7—C8	1.501 (2)
N1—C6	1.422 (2)	C8—C13	1.536 (2)
O1—C14	1.205 (2)	C8—C9	1.543 (2)
O2—C7	1.204 (2)	C8—H8A	0.9800
O3—C12	1.433 (2)	C9—C10	1.528 (3)
O3—C9	1.441 (2)	C9—H9A	0.9800
C1—C2	1.380 (3)	C10—C11	1.539 (3)
C1—C6	1.383 (2)	C10—H10A	0.9700
C1—H1A	0.9300	C10—H10B	0.9700
C2—C3	1.382 (3)	C11—C12	1.525 (3)
C2—H2A	0.9300	C11—H11A	0.9700
C3—C4	1.374 (3)	C11—H11B	0.9700
C3—H3A	0.9300	C12—C13	1.535 (2)
C4—C5	1.387 (3)	C12—H12A	0.9800
C4—H4A	0.9300	C13—C14	1.502 (2)
C5—C6	1.392 (3)	C13—H13A	0.9800
			
C14—N1—C7	111.99 (14)	O3—C9—C8	102.29 (14)
C14—N1—C6	123.71 (14)	C10—C9—C8	107.58 (15)
C7—N1—C6	124.01 (14)	O3—C9—H9A	114.2
C12—O3—C9	96.47 (12)	C10—C9—H9A	114.2
C2—C1—C6	119.77 (18)	C8—C9—H9A	114.2
C2—C1—H1A	120.1	C9—C10—C11	101.54 (15)
C6—C1—H1A	120.1	C9—C10—H10A	111.5
C1—C2—C3	120.2 (2)	C11—C10—H10A	111.5
C1—C2—H2A	119.9	C9—C10—H10B	111.5
C3—C2—H2A	119.9	C11—C10—H10B	111.5
C4—C3—C2	119.91 (19)	H10A—C10—H10B	109.3
C4—C3—H3A	120.0	C12—C11—C10	101.26 (16)
C2—C3—H3A	120.0	C12—C11—H11A	111.5
C3—C4—C5	120.8 (2)	C10—C11—H11A	111.5
C3—C4—H4A	119.6	C12—C11—H11B	111.5
C5—C4—H4A	119.6	C10—C11—H11B	111.5
C4—C5—C6	118.85 (18)	H11A—C11—H11B	109.3
C4—C5—H5A	120.6	O3—C12—C11	102.78 (16)
C6—C5—H5A	120.6	O3—C12—C13	101.63 (13)
C1—C6—C5	120.45 (17)	C11—C12—C13	110.30 (14)
C1—C6—N1	119.36 (16)	O3—C12—H12A	113.7
C5—C6—N1	120.16 (16)	C11—C12—H12A	113.7
O2—C7—N1	124.14 (17)	C13—C12—H12A	113.7
O2—C7—C8	127.30 (15)	C14—C13—C12	110.43 (14)
N1—C7—C8	108.55 (14)	C14—C13—C8	104.73 (14)
C7—C8—C13	105.51 (13)	C12—C13—C8	101.86 (13)
C7—C8—C9	112.30 (14)	C14—C13—H13A	113.0
C13—C8—C9	100.91 (14)	C12—C13—H13A	113.0
C7—C8—H8A	112.5	C8—C13—H13A	113.0
C13—C8—H8A	112.5	O1—C14—N1	124.09 (17)
C9—C8—H8A	112.5	O1—C14—C13	126.79 (16)
O3—C9—C10	103.26 (15)	N1—C14—C13	109.10 (14)
			
C6—C1—C2—C3	−0.6 (3)	C13—C8—C9—C10	74.97 (17)
C1—C2—C3—C4	0.1 (3)	O3—C9—C10—C11	31.94 (17)
C2—C3—C4—C5	0.2 (3)	C8—C9—C10—C11	−75.77 (17)
C3—C4—C5—C6	0.0 (3)	C9—C10—C11—C12	2.39 (18)
C2—C1—C6—C5	0.8 (3)	C9—O3—C12—C11	56.48 (16)
C2—C1—C6—N1	−177.25 (17)	C9—O3—C12—C13	−57.71 (15)
C4—C5—C6—C1	−0.6 (3)	C10—C11—C12—O3	−36.30 (17)
C4—C5—C6—N1	177.52 (16)	C10—C11—C12—C13	71.41 (18)
C14—N1—C6—C1	126.96 (18)	O3—C12—C13—C14	−74.48 (16)
C7—N1—C6—C1	−46.3 (2)	C11—C12—C13—C14	177.05 (15)
C14—N1—C6—C5	−51.1 (2)	O3—C12—C13—C8	36.33 (16)
C7—N1—C6—C5	135.62 (17)	C11—C12—C13—C8	−72.14 (17)
C14—N1—C7—O2	−178.77 (17)	C7—C8—C13—C14	−3.54 (17)
C6—N1—C7—O2	−4.8 (3)	C9—C8—C13—C14	113.50 (15)
C14—N1—C7—C8	−0.10 (19)	C7—C8—C13—C12	−118.62 (14)
C6—N1—C7—C8	173.85 (14)	C9—C8—C13—C12	−1.58 (16)
O2—C7—C8—C13	−179.03 (18)	C7—N1—C14—O1	176.01 (17)
N1—C7—C8—C13	2.35 (18)	C6—N1—C14—O1	2.0 (3)
O2—C7—C8—C9	71.9 (2)	C7—N1—C14—C13	−2.3 (2)
N1—C7—C8—C9	−106.67 (16)	C6—N1—C14—C13	−176.26 (14)
C12—O3—C9—C10	−54.79 (16)	C12—C13—C14—O1	−65.7 (2)
C12—O3—C9—C8	56.86 (16)	C8—C13—C14—O1	−174.64 (18)
C7—C8—C9—O3	78.51 (17)	C12—C13—C14—N1	112.55 (16)
C13—C8—C9—O3	−33.41 (16)	C8—C13—C14—N1	3.61 (18)
C7—C8—C9—C10	−173.12 (14)		

### Hydrogen-bond geometry (Å, °)

**Table:**

*D*—H···*A*	*D*—H	H···*A*	*D*···*A*	*D*—H···*A*
C10—H10B···O2^i^	0.97	2.59	3.502 (3)	156

Symmetry codes: (i) −*x*, −*y*+1, −*z*+1.

## References

[bb1] Bruker (2004). *SAINT* and *APEX2* Bruker AXS Inc., Madison, Wisconsin, USA.

[bb2] Hill, T. A., Stewart, S. G., Ackland, S. P., Gilbert, J., Sauer, B., Sakoff, J. A. & McCluskey, A. (2007). *Bioorg. Med. Chem* **15**, 6126–6134. 10.1016/j.bmc.2007.06.034 17606377

[bb3] Sheldrick, G. M. (1996). *SADABS* University of Göttingen, Germany.

[bb4] Sheldrick, G. M. (2008). *Acta Cryst.* A**64**, 112–122. 10.1107/S0108767307043930 18156677

[bb5] Wang, G.-S. (1989). *J. Ethnopharmacol.* **26**, 147–162. 10.1016/0378-8741(89)90062-7 2689797

